# A preliminary report on the feasibility of single-port thoracoscopic surgery for diaphragm plication in the treatment of diaphragm eventration

**DOI:** 10.1186/1749-8090-8-224

**Published:** 2013-12-05

**Authors:** Hsin-Hung Wu, Chih-Hao Chen, Ho Chang, Hung-Chang Liu, Tzu-Ti Hung, Shih-Yi Lee

**Affiliations:** 1 Graduate institute of mechanical and electrical engineering, National Taipei University of Technology, Taipei City, Taiwan; 2 Department of Chest, TaiTung Branch of Mackay Memorial Hospital, TaiTung City, Taiwan; 3 Department of Thoracic Surgery, Mackay Memorial Hospital, Taipei City, Taiwan; 4 Mackay Medicine, Nursing and Management College, Taipei City, Taiwan; 5 Division of Pulmonary and Critical Care Medicine, Mackay Memorial Hospital, Taipei City, Taiwan

**Keywords:** Thoracoscopy/VATS, SITS (single-incision thoracoscopic surgery), Diaphragm, Surgery

## Abstract

**Introduction:**

Thoracoscopic surgery is a popular widely used surgical technique in the treatment of common chest conditions. Conventional thoracoscopic surgery utilizes multiple small wounds for carrying out the procedure. Many procedures can also be performed with a single small port wound. In this study, we performed diaphragm plication using the techniques of single-port thoracoscopic surgery.

**Materials and methods:**

From July 1st, 2008 to December 31th, 2011, there were 21 patients admitted to our hospital due to diaphragm eventration. All of them underwent diaphragm plication. The initial 11 patients underwent two-port thoracoscopic surgery while the subsequent 10 patients underwent single-port thoracoscopic surgery.

**Results:**

The side of diaphragm eventration was on the left in all of the cases. The mean operative time was 87.3 minutes and the mean follow-up time was 17 months. There was no procedure-related complication or mortality. The time required for surgery and the postoperative pain scores were similar in the two groups.

**Conclusion:**

Single-port thoracoscopic surgery for diaphragm plication is a safe procedure. It can serve as an alternative to conventional thoracoscopic approaches to diaphragm surgery.

## Background

Diaphragm eventration is a rare condition. Most patients who are afflicted with it are asymptomatic and incidentally found in the course of health exams or the taking of a chest radiograph for an unrelated reason. However, this disease is progressive and may eventually seriously compromise lung function. The cause of disease progression results from two major reasons. The first reason is that the malfunction of phrenic nerve causing paralysis, whether complete or incomplete, of the diaphragm. The second reason is the naturally positive intra-abdominal pressure has the tendency to push the diaphragm upwards. Therefore, when the diaphragm muscle becomes incompetent, the diaphragm will be pushed upwards. It is a viscous cycle and will result in severe dyspnea if it is not adequately treated. Once the diagnosis is confirmed, therefore, the treatment is prompt diaphragm plication in order to prevent progression of disease and to maintain adequate lung function.

Conventional surgical treatment is diaphragm plication by open thoracotomy. In recent years, thoracoscopic procedures have become common in most fields of thoracic surgery. However, thoracoscopic surgery usually requires three or more port wounds to complete the procedure. In our institution, however, most thoracic surgical procedures are performed with two-port and even single-port techniques. Even in certain complex conditions, single-port thoracoscopic approach has been shown to be feasible and safe [1-6]. In this study, a variety of surgical approaches, including both two-port and single-port techniques, were used to perform diaphragm plication. We focused on the feasibility of the single-port approach and compared the outcomes the two groups.

## Methods

From July 1st, 2008 to December 31th, 2011, 21 patients were admitted to our hospital with the diagnosis of diaphragm eventration. For the initial 11 patients, we performed diaphragm plication with a two-port approach. The selection of the surgical techniques used in the study period was not randomized, because the single-port approach only became our routine practice after January in 2010. Before that time, two-port and three-port approaches were more commonly used. Therefore, the initial eleven patients underwent a two-port approach and the later 10 patients underwent a single-port approach.

**Figure 1:**
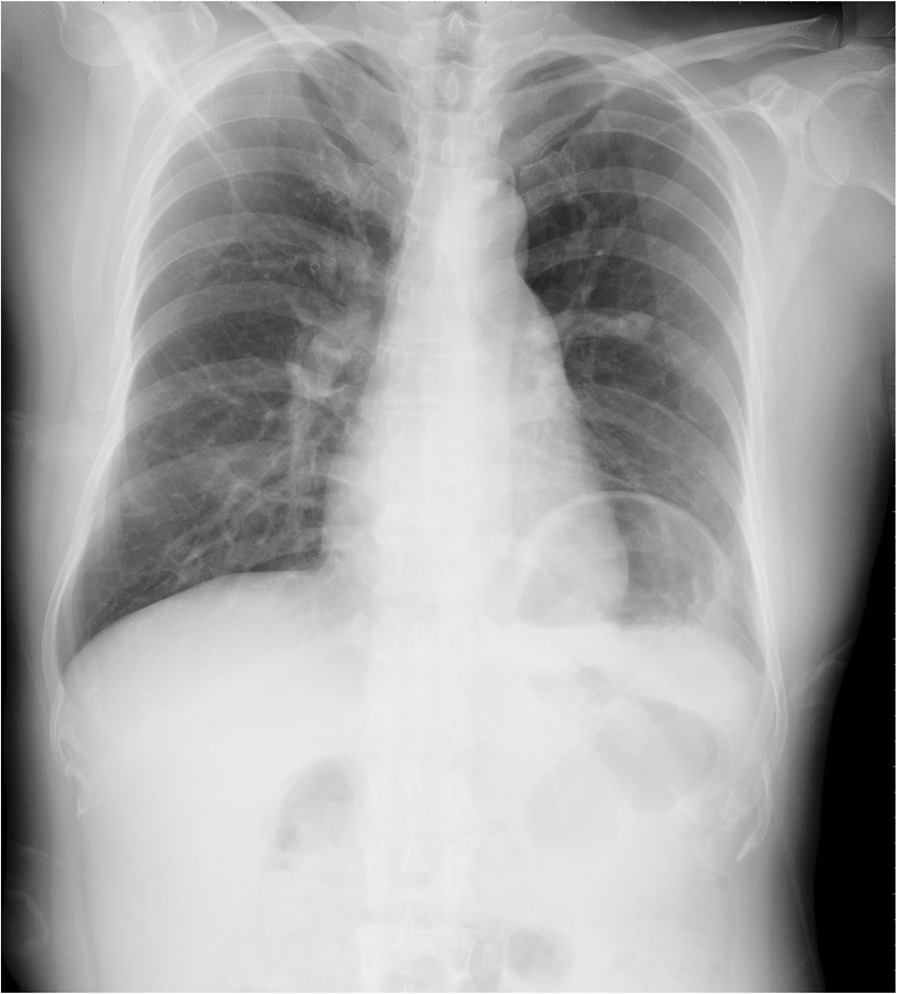
**Chest radiograph of a 44-year-old woman showed an abnormally elevated diaphragm on the left side.** Subsequent CT scan confirmed the diagnosis of eventration.

Preoperative examination included a plain chest radiograph and computed tomographic (CT) scan of the chest and routine laboratory study. The CT scan is used to exclude other causes of abnormal elevation of diaphragm, because diaphragm eventration is frequently considered to be characterized by remarkable diaphragm herniation in inexperienced staff. An example of diaphragm eventration is shown in Figure 1.

**Figure 2:**
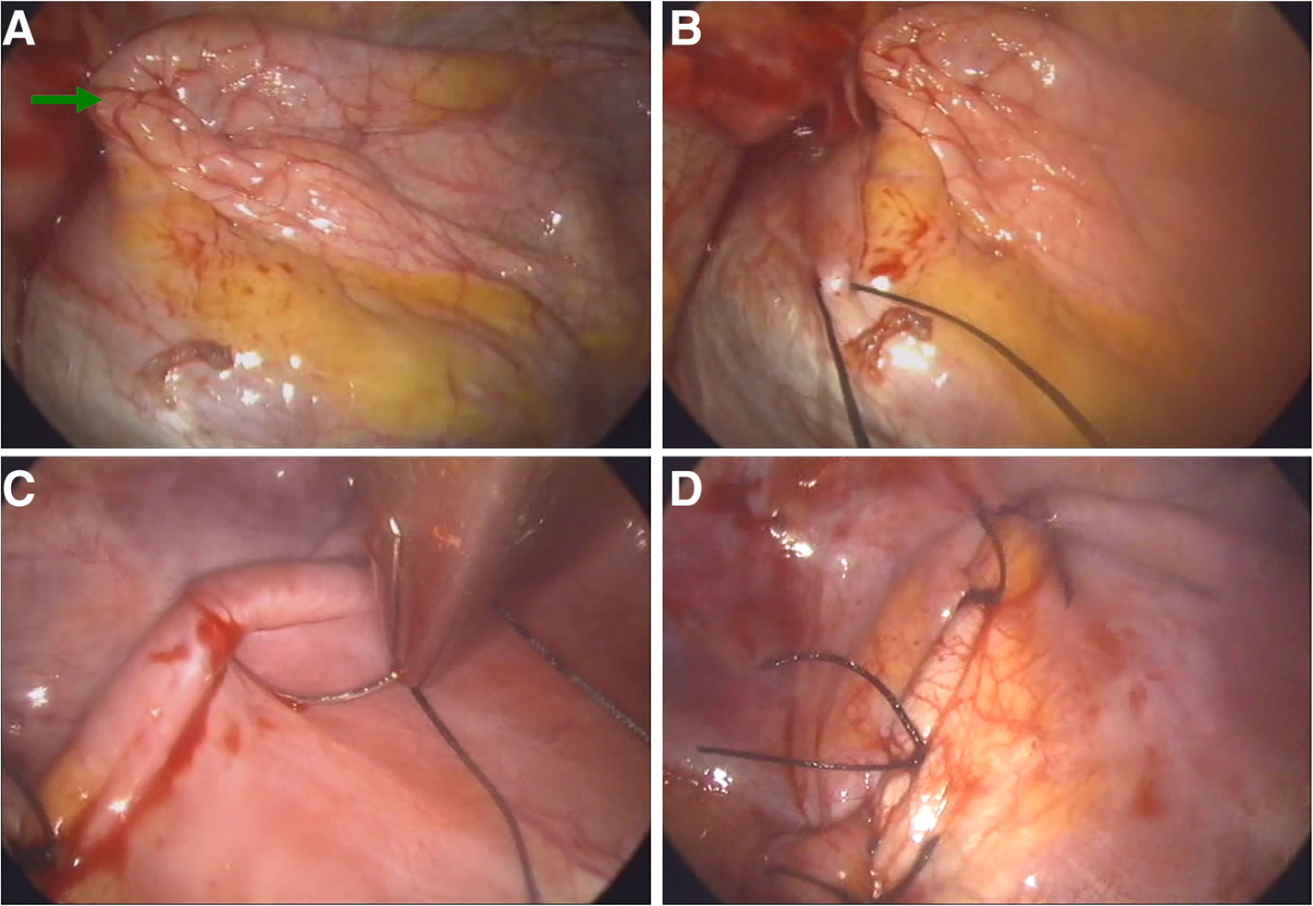
**The most flaccid part of the diaphragm can be identified under thoracoscopy (the green arrow in 2A).** After confirming the edge to be sutured, we started plication from a more medial part of the diaphragm **(B)** and then continued the procedure to a more lateral part of the diaphragm **(C)**. The overall view of the diaphragm after plication is shown in **(D)**.

The tools and instruments we used in two-port approach included a 10.5 mm thoracoscope with zero-degree field-of-view, an endoscopic needle holder and a grasper. There were two small port wounds of approximately 1.2 cm to 1.5 cm. A rigid trocar was placed in one of the port wounds and the endoscope was moved as needed in order to improve the viewing angle. Usually in the course of the procedure, one small incision was made in the 7^th^ intercostal space (ICS) along the mid-axillary line and another small incision was made in the 4^th^ or 5^th^ ICS along the anterior axillary line. We did not use a trocar in the wound for suturing. We used a 2-O silk interrupted suture for plication. The most flaccid part of the diaphragm was identified (Figure 2A) and then we introduced the sutures from diaphragm near the mediastinum (Figure 2B) to the most lateral part of diaphragm (Figure 2C and 2D). At the end of the procedure, we placed one 24Fr to 28Fr. chest tube. The tube was removed one to two days after the operation. Patients were discharged, after which they were followed in the outpatient department.

**Figure 3:**
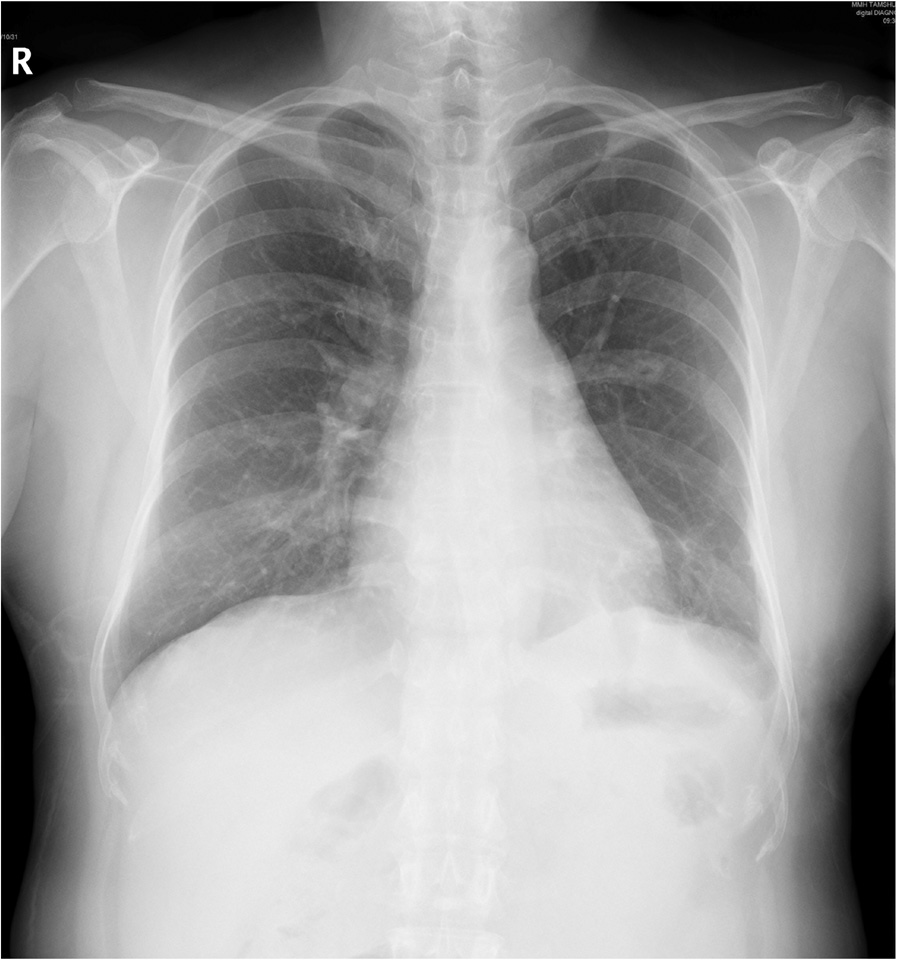
**Forty-eight hours after the operation.** The diaphragm (showed) exhibits a normal position after we removed her chest tube.

In the performance of the single-port approach, the endoscopic tools we used included a 5-mm endoscope with a 30-degree field-of-view, a needle holder and a grasper. The wound size was 1.5 cm to 2.0 cm and it was made in the 6^th^ ICS along the anterior axillary line. Because there was only a small incision, we avoided the use of rigid trocar. The endoscope, needle holder and grasper were utilized in the same working space. The endoscope was kept in the upper wound edge in order to see the procedure clearly without hindering suture. The procedure of suture plication was performed essentially the same as in the two-port approach. At the end of the procedure, we placed a chest tube from 24 Fr to 28 Fr. The procedures of removing the chest tube, discharge and follow-up are the same as in the two-port approach. In all of the treated patients, we injected 1 ml to 3 ml Marcaine as an intercostal block in the soft tissues near the intercostal nerve. The endotracheal tube was removed immediately after the operation. In the recovery room, all of the patients had a portable chest radiograph taken to confirm the adequacy of the plication (Figure 3).

In the case of all of the patients, the time required for the operation, hospital stay, visual analogue scores for pain, outcomes and follow-up period were recorded to allow for comparison. We used SPSS 13.0 for the Student *t*-test. A p-value less than 0.05 was considered as significant.

## Results

There were 21 patients included in the study. All of them had an abnormally elevated diaphragm on the left side. There were only five patients who had a minor chest contusion in the past and there was no patient who had undergone surgery of the chest or abdomen. The clinical data is shown in Table 1. The mean age was 54.9 years old. There were 15 women and 6 men. The average time required for the operation was 87.3 minutes. The mean follow-up period was 16.9 months.

**Table 1:** Clinical demographics and outcomes of all patients undergoing thoracoscopic diaphragm plication

		**Two-port**	**Single-port**	**p-value**
NO		11	10	
Age (years)		54	55.8	0.83
Sex	Men	3	3	0.89
	Women	8	7	
Side	Right	0	0	
	Left	11	10	
OP time (min)		92.2	82	0.25
F/U (months)		19.6	14	0.11
HS (days)		3	2.85	0.53
VAS	24-hour	4.1	3.85	0.57
	36-hour	3.3	3.5	0.48
PSD	Improved	8	8	
	No change	3	2	0.67
	Worse	0	0	

NO: number, OP time: time required for operation, F/U: follow-up, HS: hospital stay, VAS: visual analogue scores for pain, PSD: postoperative symptom of dyspnea.

In two-port group, the mean age was 54 years. Eight women and three men were operated for left sided diaphragm eventration. The average time required for the operation was 92.2 minutes. The average pain scores at 24 hours and 36 hours after the operation were 4.1 and 3.3, respectively. In the single-port group, the mean age was 55.8 years old, slightly older than that in the two-port group, but the difference was not significant (*p* value: 0.83). There were 7 women and 3 men in the single-port group. The mean time required for the procedure was 82 minutes, slightly faster than that in the two-port group, but again, the difference was not significant (*p* value: 0.25). The pain scores at 24 hours and 36 hours after the operation were 3.85 and 3.5, respectively. The pain scores in the single-port group were slightly better than the two-port group, but the difference was not significant. The p-value of the pain scores at 24 and 36 hours were 0.57 and 0.48, respectively. The hospital stay of the patients in the two groups was similar (*p* value: 0.53).

There was no surgical mortality and all patients are still alive and have had no recurrence. The overall results showed that the patient outcomes were similar. Hospital stay, pain scores and time required for the operation did not significantly differ between the groups.

## Discussion

Diaphragm eventration is a rare condition. Although the natural course is indolent, the disease is nevertheless progressive. Some of the patients may be diagnosed as having diaphragm herniation. In difficult cases, a huge diaphragm herniation can appear to be very similar to eventration on a chest radiograph. However, further investigation, usually with a CT scan, can help establish the correct diagnosis prior to the operation. A variety of causes had been proposed to account for its etiology. However, regardless of the cause, the mainstay of treatment is surgery.

Conventional thoracoscopic surgery for diaphragm plication typically utilizes three small port wounds. Each endoscopic instrument needs one port wound, i.e. the thoracoscope, grasper and needle holder. With 3 small incisions, surgeons can easily create a 3-dimensional working environment and better field-of-view. The instruments and endoscope can change to the other port wounds as needed for a better orientation and to avoid a difficult angle of approach. Our team has utilized a variety of thoracoscopic procedures using a two-port approach since 2008. Due to increased feasibility and technical improvement, as well as growing familiarity of the team staff, a single-port approach became our first-line thoracoscopic procedure except for certain types of emergency operations and open thoracotomy. A variety of diseases in the chest have been successfully treated with the single-port approach, including lung cancer resection, radical lymph node dissection, esophagectomy with reconstruction (along with a laparoscopic approach), decortication, wedge resection of the lung for biopsy, and pneumothorax treatment and diaphragm repair with a very low conversion rate [1,3,6-8]. In designing diaphragm surgery for specific cases, some technical modifications should be considered as needed.

### Proper port wound location

The port wound is confined to a certain site, which may make manipulation of the endoscopic instruments difficult. According to our experience, the most appropriate location for diaphragm surgery is in the 6^th^ ICS along the anterior axillary line [8]. The extent of the condition severity may make the procedure more complicated. The 6^th^ ICS is usually appropriate for a better field-of-view and suturing. The endoscope had better be placed in one of the wound edge, usually in the upper edge. Then the grasper is placed in lower wound edge. The space of the middle part can allow suturing. If needed, the 5^th^ ICS and 7^th^ ICS can serve as alternatives, depending on the severity of eventration and the patient’s type of build. The reason we choose a more anterior location rather than a more posterior location is due to the wider ICS in the anterior aspect. For a similar incision in a more posterior aspect, manipulation of the endoscopic instruments and endoscope will be more difficult due to very narrow ICS, especially when using a single-port approach. In contrast, in anterior ICS, manipulation of the instruments and arrangement of the endoscope is easier. Interference between a regular straight endoscope and instruments may be unavoidable. As a result, the endoscope should always be placed in upper wound edge to ensure an adequate field-of-view.

### Suture techniques

**Figure 4:**
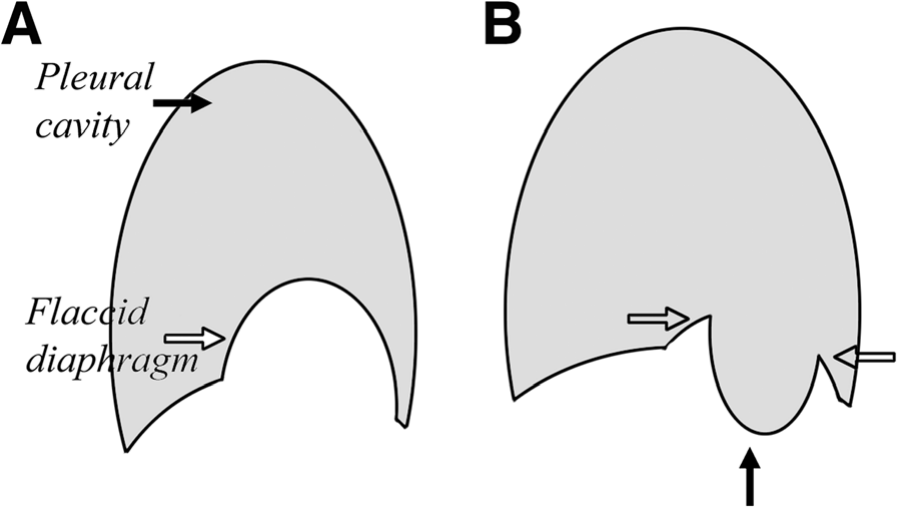
**Once we identified the most flaccid part of the diaphragm (the hollow arrow in A), we used the instruments to push the intra-abdominal organ downwards, after which the proper edge to be sutured could be identified (the hollow arrow in B).** Usually, the intra-abdominal organs which needed to be pushed downwards did not come upwards to impede subsequent sutures.

**Figure 5:**
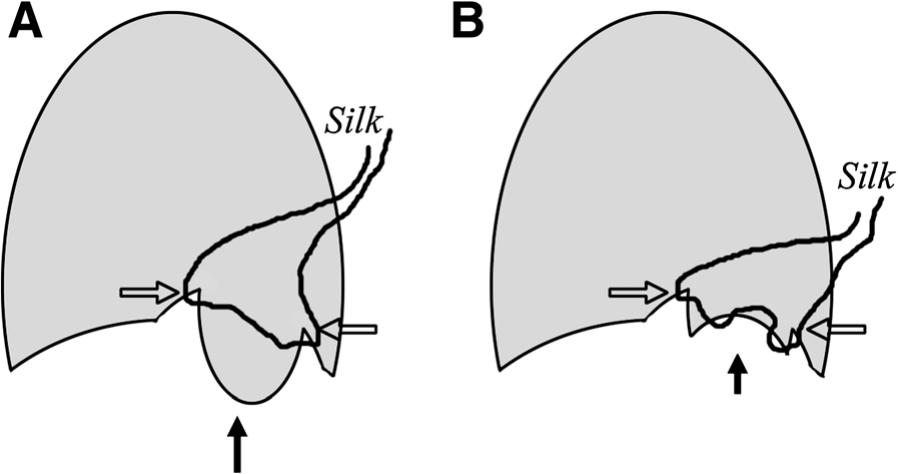
**Usually, suturing the edge that was identified in the previous step is sufficient for plication (A).** Interrupted sutures were performed from the medial part to lateral region. In the case of a severely flaccid diaphragm, multiple layers of the diaphragm have to be sutured to achieve secure plication **(B)**.

The first step after the small incision has been made is to identify the flaccid part of the daiaphragm (Figure 4A). Then it is necessary to identify the appropriate edge to be sutured (arrows in Figure 4B). Because diaphragm plication in such a condition should be permanent, the suture materials should be of large bore and not be of a type to become degraded spontaneously. In all of the cases we treated, the suture was made of silk. Both the interrupted and continuous methods are acceptable for proper plication. If the flaccid and elevated diaphragm is homogenous, plication can be done in any part of the diaphragm as long as the phrenic nerve is not injured. In cases in which the diaphragm has an obviously flaccid portion, plication should be performed in that region to restore adequate tension (Figure 1A). Depending on the surgeon’s preferences, plication may be started from a more lateral part to medial part of the diaphragm. Based on our experience, suturing from a more medial part of the diaphragm is generally easier. An interrupted suture may be safer to avoid dehescience, but it is also more time-consuming. The tying of each suture was done extracoporeally and then a pusher was used to secure the knot onto the diaphragm. The sutures were performed one by one (Figure 5A). With such a modification, the procedure can actually be faster than tying all the knots inside the pleural space. In the case of a very flaccid diaphragm, the sutures may include multiple layers of the diaphragm in one knot, as shown in Figure 5B. In any case, the phrenic nerve should be carefully protected from injury. Protection of the intra-abdominal organs should be secured during the procedure. When needed, we may use Allis clamp to pick-up the diaphragm before suture in order to avoid unexpected injury to the intraabdominal organs. In performing continuous running sutures, the scope was placed in the wound edge while allowing maximal space for manipulation of the needle holder. After the first knot is secured, we pull the silk extracoporeally to test for the tension and consistency from the medial to the most lateral part of the flaccid diaphragm.

### Minimum effective wound size

With the continuing development and maturation of certain thoracoscopic surgical techniques, the issue of the theoretically smallest wound size should be discussed. A variety of thoracic surgery procedures involve the extraction of a specimen at the final step. The size of the specimen may be a factor that helps determine the minimum effective wound size. We previously proposed a concept of minimum effective wound size in a prior study of single-port thoracoscopic surgery [2,5,6,8-10]. However, there is no specimen that needs to be removed in the procedure of diaphragm plication. The nature of the procedure may therefore challenge conventional concepts of wound size. From our experience, the only absolutely limiting factor is the overall size of instruments. The overall size of the needle holder, grasper and endoscope itself is roughly 1.5 cm. This could well be the effective size limit for the smallest incision.

The limitations of the current study include the small number of cases and relatively short period of follow-up. All patients in the study had left side disease, which is easier to perform the surgery. We could not extend the limited experiences to the right side disease. Comparison of the two groups may be confounded by the potential learning curve since these cases are sequential rather than randomized. Although as yet there is only preliminary experience, the techniques we have proposed may be a feasible and reasonable alternative approach for treating diaphragm abnormalities.

## Conclusion

Diaphragm plication using a single-port thoracoscopic approach is both feasible and safe. It can serve as an alternative approach to conventional multi-port thoracoscopic procedures for diaphragm surgery.

## Abbreviations

NO: Number; OP time: Time required for operation; F/ U: Follow-up; HS: Hospital stay; VAS: Visual analogue scores for pain; PSD: Postoperative symptom of dyspnea.

## Competing interests

Dr. HH Wu, CH Chen, professor H Chang, Dr. HC Liu, Ms. TT Hung, and Dr. SY Lee have no competing interests.

## Authors’ contributions

HHW and SYL are the doctors for the primary care of the patients. CHC and TTH wrote the article. HCL and CHC performed the surgery and assisted for the postoperative care. HC is the supervisor of performing the study. All authors read and approved the final manuscript.
